# We know CBT-I works, now what?

**DOI:** 10.12703/r/11-4

**Published:** 2022-02-01

**Authors:** Alexandria Muench, Ivan Vargas, Michael A Grandner, Jason G Ellis, Donn Posner, Célyne H Bastien, Sean PA Drummond, Michael L Perlis

**Affiliations:** 1 Behavioral Sleep Medicine Program, Department of Psychiatry, University of Pennsylvania, PA, USA; 2 Chronobiology and Sleep Institute, Department of Medicine, University of Pennsylvania, Philadelphia, PA, USA; 3 Department of Psychological Sciences, University of Arkansas, Fayetteville, AR, USA; 4 Department of Psychiatry, University of Arizona, AZ, USA; 5 Northumbria Centre for Sleep Research, Northumbria University, UK; 6 Department of Psychiatry & Behavioral Sciences, Stanford University, Stanford, CA, USA; 7 School of Psychology, Laval University, Pavillon Félix-Antoine-Savard, Québec, Canada; 8 Turner Institute for Brain and Mental Health, School of Psychological Sciences, Monash University, Victoria, Australia

**Keywords:** CBT-I, Insomnia, Efficacy, Future Directions

## Abstract

Cognitive behavioral therapy for insomnia (CBT-I) has been shown to be efficacious and now is considered the first-line treatment for insomnia for both uncomplicated insomnia and insomnia that occurs comorbidly with other chronic disorders (comorbid insomnia). The purposes of this review are to provide a comprehensive summary of the efficacy data (for example, efficacy overall and by clinical and demographic considerations and by CBT-I formulation) and to discuss the future of CBT-I (for example, what next steps should be taken in terms of research, dissemination, implementation, and practice).

In this article, we review the evidence regarding the efficacy of cognitive behavioral therapy for insomnia (CBT-I) and answer the question “Now what?” The review is meant to be comprehensive and presents not only summary data regarding treatment outcomes and durability but also what is known about the efficacy of CBT-I in real-world practice and in comorbid insomnia and how CBT-I outcomes vary with race, sex, and age and by delivery format. In addition, information on two prominent adaptations to CBT-I is presented: (1) briefer forms of CBT-I and (2) the adoption of mindfulness as an adjuvant therapy. The “Now what (what’s next)?” section highlights prior commentaries on this subject and adopts a Q-and-A format to provide answers to questions pertaining to what’s next for insomnia treatment research. These issues span the gamut from what clinical research is needed to asking and answering questions relevant for professional practice (that is, guidelines and policy, dissemination and implementation, and practice issues).

This article was inspired by a symposium session of the same name at the Associated Professional Sleep Societies in 2019. Four presentations were given at that time: one reviewed behavioral sleep medicine (BSM) field leadership’s recommendations regarding CBT-I (Michael Perlis), one regarded the relevance of (and how to measure) adherence during CBT-I (Sean Drummond), one reviewed what is known about alternative delivery modes (Simon Kyle), and one regarded the need for the adaptation of CBT-I to “real-world challenges” (Bei Bei). The present work does not necessarily represent the views presented by the lecturers at that time but is certainly informed and inspired by them. Given that symposia and this article, perhaps what stands out most is that we are far from being ready to “rest” on our laurels^1–3^.

## CBT-I works

### The efficacy of CBT-I 

There is now an overwhelming preponderance of evidence that in-person CBT-I is effective^4–14^, as effective as sedative-hypnotics during acute treatment (4–8 weeks)^6,9,15^, and is more effective than sedative-hypnotics in the long term (for example, more than 3 months after treatment)^13,14^. This overall profile, along with the low propensity of CBT-I for side effects or harm, is likely what prompted the American College of Physicians to recommend that CBT-I be considered the first-line treatment for chronic insomnia^16^. This position has since been adopted by the European Sleep Research Society^17^, the Australasian Sleep Association^18^, and the Department of Veterans’ Affairs/Department of Defense (VA/DOD)^19^.

***Pre-to-post treatment change (overall).*** In terms of symptom reduction (pre-post treatment change), subjective sleep latency (SL) and wake after sleep onset (WASO) times are reduced from baseline averages of about 60 minutes to about 30 minutes at treatment end (Table 1). These absolute changes correspond to average treatment effects of about 50% reductions in symptom severity and pre-to-post effect sizes of about 1.0^4,12,14^. Early morning awakenings (EMAs) are generally collapsed into WASO measures and therefore little is known about the effects of CBT-I on EMA. In regard to total sleep time (TST), this measure has been reliably found to be the least affected by CBT-I^6,12,20^; only about 45% of patients exceed baseline TST at treatment end^21^. In the context of acute treatment, mean changes in TST are generally less than 30 minutes, pre-post change is less than 10%, and within-subject effect sizes are less than 0.5^22–24^.

**Table 1.**
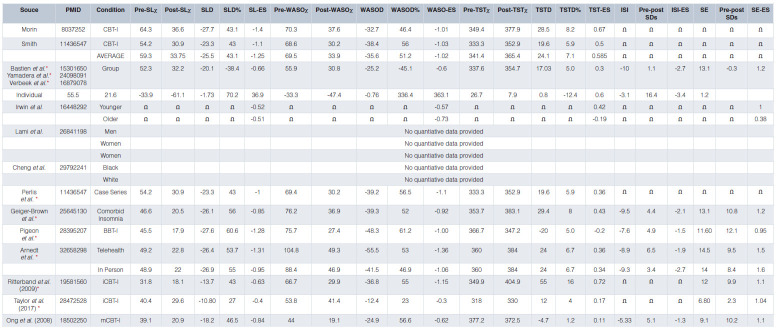
CBT-I Outcomes Overall and by Variable Group or Type. *, ES calculated; Ω, Insufficient Data (ESs not avaiable, nor the data required for their calculation). Note: The missing data above should in and of themselves consitute a road map for work that needs to get done.

One final point in regard to TST: although it stands to reason that the sum of SL + WASO (that is, total wake time or TWT) decreases should equal TST gains, this is often not true, for two reasons. First, with sleep restriction, the change in TIB limits potential gains in TST. Second, patients rarely calculate TST but instead provide their impression of how much sleep was obtained. That is, given TIB and SL, WASO, and EMA, it is possible to estimate TST in a manner that is not a “guesstimate” but instead may be arithmetically calculated in a manner that is internally valid; that is, TST = TIB – (SL + WASO + EMA). Given these two considerations, it is rarely the case that the decrease in TWT equals the increase in TST. When treatment outcome is assessed with retrospective estimates of overall illness severity (before and after treatment by using instruments such as the Insomnia Severity Index [ISI]^25^), the effect sizes are reliably larger than single measures of sleep continuity, and within-subject effects sizes are around 2.0^12^. This is not surprising as the ISI assesses not only illness severity (magnitude of sleep continuity disturbance) but also the degree of insomnia-related daytime impairment/complaint. When evaluated in terms of percentage of patients who exhibit treatment responses^20^ (typically defined by using the ISI), between 70 and 80% of patients achieve a therapeutic response during acute treatment^20,26^.

***Durability of CBT-I effects over time.*** In what are perhaps the first long-term randomized controlled trials (RCTs) of CBT-I, it was found that SL and WASO effects are remarkably stable over time periods of up to 24 months^20,23^. This is to say that clinical gains are maintained for months and years after treatment is discontinued. Interestingly, TST effects (which are initially marginal) appear to accrue with time. That is, when followed longitudinally, patients exhibit an average increase in TST of about 50 minutes (about 12 minutes per measurement interval: 3, 6, 12, and 24 months)^20,22,23,26^. These gains do not appear to be related to additional improvements in SL and WASO but instead are likely to be related to increased time in bed while maintaining good sleep efficiency. When evaluated in terms of percentage of patients who exhibit remission, 50 to 60% of treatment responders achieve remission in the 6 to 12 months that follow therapy. Similar findings were recently presented for a large-scale clinical case series study^27^. In that study, mean ISI values from end of treatment (T1) to follow-up (T2 [4–10 years]) were found to be remarkably stable (baseline ISI score 17.1 ± 4.5, T1 = 9.7 ± 4.6, and T2 = 9.9 ± 6.3). In contrast to these studies, a recent meta-analysis on the durability of CBT-I showed that CBT-I continues to be effective at 3, 6, and 12 months as compared with non-active controls but that the clinical gains in the active-treatment group appeared to decline over time^28^. Although these differences in claims remain to be reconciled, it may be the case that the meta-analytic findings differ owing to between-study differences in the application of CBT-I. If protocol differences yield different long-term outcomes and the individual studies summarized here used the most robust methodologies, then the durability outcomes in the meta-analysis may represent the inclusion of studies with small-magnitude outcomes and/or greater variability in the effect size estimates. Given the absence of end-of-treatment effect sizes in the meta-analysis, one cannot be sure whether such data were comparable to the single studies summarized here or other meta-analytic studies regarding the acute effects of CBT-I.

### The efficacy of “real world” CBT-I (RCTs vs. clinical case series studies)

The conclusions that can be drawn from RCTs are often subject to some skepticism. Many believe that RCT outcomes represent the best-case scenario and are not generalizable to clinical practice. This perspective is rooted in the reasonable belief that RCTs are populated by exceptionally healthy patients (individuals who have the illness of interest but little else in the way of comorbidity) but that in-clinic patients often have complex medical, psychiatric, and psychosocial profiles and therefore may be less responsive to targeted treatment. Nonetheless, this scenario need not be true. For example, in a secondary analysis, it was found that subjects who completed at least a minimum dose of CBT-I (at least four sessions) experienced about a 23-minute decrease in SL (d = 1.00), a 39-minute decrease in WASO (d = 1.09), and a 20-minute increase in TST (d = 0.36)^29^. These outcomes appear to be comparable to those observed in RCTs.

In sum, it may be the case that “real world” patients benefit from CBT-I to the same extent as subjects in clinical trials, perhaps more. This surprising result, if true, may be ascribable to a variety of factors, including better outcomes due to professional therapists, the tailoring of the clinical treatment regimen to the individual case, and other non-specific factors like cognitive dissonance (for example, paying for treatment vs. no payment or being paid to receive treatment).

### Efficacy of CBT-I in comorbid insomnia

By and large, the effects summarized above are from foundational RCTs that were undertaken from 1990 until the early 2000s. The overwhelming majority of these trials were conducted in subjects with “primary insomnia” (that is, individuals without comorbid illnesses that include insomnia as a symptom). This was the case because insomnia was construed as both a symptom and a disorder, and it was believed that so-called “secondary insomnia”, now known as comorbid insomnia, could be effectively treated only with therapies for the primary disorder^30^. For example, insomnia occurring in the context of chronic pain would be ameliorated only to the extent that the analgesic therapy was successful. Implicit in this perspective was that direct treatment of insomnia, in the context of chronic pain, would yield little to no positive effects, as pain (not sleep extension) was considered to be the perpetuating factor for this form of insomnia. In the last two decades, dozens of CBT-I RCTs have been conducted in patients with comorbid insomnia (including but not limited to patients with depression^31,32^, bipolar disorder^33^, post-traumatic stress disorder [PTSD]^34^, generalized anxiety disorder^35^, schizophrenia^36^, cancer^37^, heart failure^38^, chronic pain^39^, Alzheimer’s disease^40^, multiple sclerosis^41^, alcoholism^42^, chronic obstructive pulmonary disease^43^, obstructive sleep apnea^44^, and period limb movement disorder^45^). To the best of our knowledge, all of these studies found that CBT-I was effective. Most studies found that the treatment outcomes were similar to those observed in patients with “primary” insomnia, and several studies found superior treatment outcomes^28–42^. At least one clinical case series study was conducted on this topic and there have been several meta-analyses^46^. Geiger-Brown *et al.* summarized 37 studies (1,379 subjects), where SL decreased by 26 minutes (d = 0.85), WASO decreased by 39 minutes (d = 0.92), and TST increased by 29 minutes (d = 0.43). Moreover, there was a 13-point improvement in SE (d = 1.20) and about a 10-point increase on the ISI (d = 2.1)^46^. These treatment effects were found to be durable for up to 18 months post-treatment. Not only do these data demonstrate the efficacy of CBT-I with insomnia in the context of comorbidities, but now an accumulating evidence base shows that treating insomnia with CBT-I has a beneficial “halo” effect on other medical and behavioral health conditions. For example, CBT-I as an augmenting strategy to the medical management of depression has been found to double antidepressant treatment response and to reduce suicidality by half^47,48^. Moreover, numerous studies suggest that CBT-I can be protective against the development of depression in people with insomnia, which is important given the high concordance between insomnia and depression^49–54^. Most recently, Irwin *et al.*, in perhaps the most persuasive demonstration to date, showed that CBT-I in patients with recurrent depression prolonged remission rates and nearly halved the rate of new-onset depression as compared with a control condition^55^. Finally, a similar set of outcomes were found for internet-based CBT-I (iCBT-I) as compared with internet-based CBT for depression (iCBT-D)^56^, where only insomnia treatment produced positive insomnia outcomes, but both forms of iCBT produced positive depression outcomes.

Despite numerous studies showing the efficacy of CBT-I in patients with comorbid insomnia, the effect sizes in the meta-analyses tend to be smaller than the effect sizes of RCTs in those with uncomplicated insomnia. Unless this is an artifact of the aggregation of data over RCTs with vastly differing methodologies, it may be genuinely the case that CBT-I in patients with comorbid insomnia produces fewer treatment responders but that the response itself is of a normal magnitude. This possibility may be related, in part, to how adherence varies from clinical population to clinical population. In subjects with uncomplicated insomnia, their entire focus may be on the insomnia and its treatment. In subjects with comorbid insomnia, some of the their focus may be on the comorbid disorder and this may limit their ability to engage in, and benefit from, CBT-I. In other words, owing to state-wise exacerbation of symptoms from comorbid disorders, it may be harder to garner adherence day to day. This is an inference from the data that exist; to the best of our knowledge, no studies have compared uncomplicated and comorbid insomnia in regard to adherence and whether adherence is less when the severity of comorbid illness is worse. If true, this may account for differential responder rates.

### CBT-I outcomes by race, sex, age

***CBT-I outcomes by race.*** Most CBT-I trials have study samples that predominately are white^57^. When minorities have been included, there was usually not sufficient representation of any particular race or ethnic group to allow for post hoc assessment of whether CBT-I outcomes vary by race/ethnicity^58^. There is, however, one exception: a study by Cheng *et al.* from 2018^49^. That study was an assessment of the efficacy of an internet-based, six-session CBT-I protocol in 658 individuals (140 of whom were African-American and 37 of whom were designated as “other race/ethnicity”). The researchers concluded, on the basis of a secondary analysis, that there were no racial differences in regard to treatment outcome or attrition. This finding may be due to a ceiling effect with online CBT-I. That is, all CBT-I interventions that successfully do the minimum will produce significant pre-to-post change (not much less and not much more). Under these circumstances, one is not likely to observe interactions of any kind (by race/ethnicity, by age, by sex, and so on). Nonetheless, it is important that future studies embrace race as a relevant factor (*a priori*).

***CBT-I outcomes by sex.*** Despite the common finding that women are nearly twice as likely as men to develop acute and chronic insomnia^59,60^, there has not been a dedicated line of research probing why, compared with men, women are more vulnerable to insomnia or whether the two sexes differ in regard to adherence or treatment outcome. To the best of our knowledge, only one study has looked at sex differences in individuals with diagnosed fibromyalgia undergoing CBT-I (n = 28)^61^. The outcome variables of interest were SL and SE as measured with the Pittsburgh Sleep Quality Index^61^. There were no significant pre-to-post treatment differences between sexes. Sleep continuity data as measured prospectively with high-density sampling (that is, sleep diaries) were not obtained in that study.

In contrast to the evaluation of by-sex interactions in patients with insomnia disorder or comorbid insomnia, efficacy studies of CBT-I as applied to insomnia occur in the context of women’s reproductive health (pre-menstrual dysphoric disorder, pregnancy, post-partum, and menopause)^62–71^. Two such studies are discussed below.

In regard to pregnancy, research suggests that women are more predisposed to insomnia and subsequent depression during pregnancy and the post-partum period. Swanson *et al.* (2013)^71^ conducted CBT-I over five sessions in 12 women who were post-partum and who endorsed clinically significant levels of sleep continuity disturbance, as measured by sleep diaries and the ISI. Sleep improved significantly over the course of treatment where WASO decreased from 124.8 to 49.42 minutes (d = −1.87), TST increased from 382.8 to 419.0 minutes (d = 0.78), SE% increased from 74.9 to 89.3% (d = 1.97), and the ISI score decreased from 17.5 to 7.8 (d = −2.06).

With regard to menopause, research suggests that CBT-I is efficacious in both menopausal and peri-menopausal women. Specifically, McCurry *et al.* (2016)^62^ conducted an RCT in both menopausal and peri-menopausal women, who were randomly assigned to receive either 8 weeks of telephone-based CBT-I or menopause education (n = 53 in each group). Significant differences were found between the treatment and control conditions for SL, WASO, TST, SE%, and the ISI. The pre-post change in the treatment group was as follows: SL decreased from 54.4 minutes at baseline to 22.9 minutes at 8 weeks (a change of −31.5 minutes; confidence interval [CI] −39.2, −23.8), WASO decreased from about 71 minutes to about 34 minutes (a change of −37.4 minutes; CI −48.3, −26.6), and, not surprisingly, there was minimal change in TST, which increased from about 6.5 hours to about 7 hours (a change of 0.4 minutes; CI −0.1, 0.9). Differences were also found for the ISI score and SE%. The ISI score decreased from about 15 to about 5 (a change of −9.9 points; CI −11.2, −8.7), and SE% increased from about 75.8 to about 87% (a change of 12.1%; CI 9.4, 14.8).

***CBT-I outcomes by age.*** At least three meta-analyses suggest that behavioral interventions, including CBT-I, are effective in reducing insomnia severity across the life span^7,8,72^. One of the most comprehensive of these meta-analyses was conducted by Irwin *et al.* in 2006^8^. In that review, 23 RCTs of behavioral interventions in middle-aged (<55 years) and older (>55 years) adults were reviewed. Behavioral interventions were grouped into three categories: “omnibus CBT-I” (that is, behavioral, cognitive, and imagery components), relaxation treatments (that is, progressive muscle relaxation, biofeedback, and hypnosis), and behavioral-only interventions (that is, stimulus control therapy [SCT] and “sleep compression”). Middle-aged adults showed greater improvement than older adults in TST (d = 0.42 vs. d = −0.19, respectively) and SE (d = 1.00 vs. d = 0.38, respectively). In contrast, there were minimal differences for SL (middle-aged adults, d = −0.52; older adults, d = −0.51) and in WASO (middle-aged adults, d = −0.57; older adults, d = −0.73). Given the high prevalence of insomnia in older adults, it stands to reason that future research should focus on optimizing TST (for example, ensuring that the patient is getting a TST that is optimized to their age and preference). TST is commonly found to be the least affected sleep continuity variable, regardless of age^20^.

### CBT-I in Alternative Formats

***CBT-I delivered in group format.*** Although it may seem that the delivery of CBT-I in group format is a relatively new development, this is not the case. In fact, the first insomnia treatment studies conducted in groups were in 1974 (although these were evaluations of relaxation treatment)^73^. The first study with SCT delivered in groups was in 1979^74^. Thus, contrary to what one might imagine, group CBT-I has been a standard mode of treatment delivery since the first insomnia RCTs, although it was rarely featured as a relevant detail (as an alternative to individual treatment). This naturally leads to the question, “Do the two formats produce different outcomes?” To the best of our knowledge, there have been three head-to-head trials: one by Bastien *et al.* (2004)^75–77^, one by Verbeek *et al.* (2005)^75–77^, and one by Yamadera *et al.* (2014)^75–77^. Pre-post means and average effect sizes were calculated for all three studies (Table 1). Averaging affects across trials, Bastien *et al.* (2004), Verbeek *et al.* (2005), and Yamadera *et al.* (2014) found that the group interventions produced about a 20-minute decrease in SL (d = −0.7), a 33.3-minute decrease in WASO (d = −0.6), a 26.7-minute improvement in TST (d = 0.3), and about a 16.4% increase in SE (d = 1.2). (SE and WASO averages were calculated from only Bastien *et al.* and Verbeek *et al.* as Yamadera did not report these outcomes.) Averaging affects across trials, Bastien *et al.* (2004), Verbeek *et al.* (2005), and Yamadera *et al.* (2014) found that the individual interventions produced about a 33.9-minute decrease in SL (d = −1.7), a 25-minute decrease in WASO (d = −0.8), a 17-minute improvement in TST (d = 0.8), and about a 13% increase in SE (d = 1.2). Interestingly, all three investigations found that group CBT-I produced poorer outcomes for sleep initiation (that is, less robust effects on SL) than did individual treatment. Although the reasons for this difference are unknown, it has been speculated that anxious patients (to the extent that this is more typical of sleep onset problems) may fare less well in group treatment owing to social anxiety and that socially anxious individuals may be less likely to engage in group treatment and this serves to create a ceiling effect.

***CBT-I delivered as via telehealth (video conferencing).*** Telehealth CBT-I represents one substantial way to extend the catchment area of individual clinicians. Not surprisingly, the first forays into this delivery modality were conducted within the VA health system. This was likely the case because the VA is a national health-care program that is not restricted by state licensing. That is, a CBT-I therapist in any given state may see VA patients at any VA facility, nationwide. This allowance likely prompted the VA to deploy telehealth earlier than most health-care systems. In recent years, more so now because of the Covid-19 pandemic, HIPPA (Health Insurance Portability and Accountability Act)-compliant videoconferencing is more widely available. More specifically, many states are allowing for telehealth assessment and interventions across state boundaries as a way of meeting the population’s increased health-care needs. Whether such exemptions will continue into the post-pandemic era is unknown. Nonetheless, there are new initiatives to promote the interstate provision of health services, such as the Psychology Interjurisdictional Compact (PSYPACT). Leaving aside the regulations that restrict access to telehealth CBT-I, there is the question of its relative efficacy. To date, one study has systematically evaluated CBT-I via videoconferencing versus in-person CBT-I. Arnedt *et al.* (2020) conducted this RCT in 65 adults with chronic insomnia^78^. Subjects were randomly assigned to either six-session telemedicine or in-person CBT-I. It was found that SL decreased by about 26 minutes (d = −1.31) in the telemedicine group and by about 27 minutes (d = −0.95) in the in-person arm; WASO decreased by 55 minutes in the telemedicine group (d = −1.36) and 42 minutes in the in-person arm (d = −1.06). Interestingly, TST did not differ by group. Both groups decreased by 24 minutes (d = 0.36). Outcomes on SE% and the ISI were also similar across the two groups. SE increased for both groups by about 14% (telemedicine d = 1.5 and in-person d = 1.6). The ISI score decreased by about 9 points for both groups (telemedicine d = −1.9 and in-person d = −2.7). In sum, it would appear that telehealth CBT-I is at least non-inferior to in-person CBT-I (that is, the two delivery modalities do not meaningfully differ). To find otherwise, to our way of thinking, would be surprising in that both delivery modalities allow for many of the positive attributes of in-person therapy, such as the monitoring of non-verbal cues and the presentation of visual aids as needed (the presentation of white board exemplars, figures, and so on).

***Online CBT-I (iCBT-I [unattended-automated “apps”]).*** In the last decade, internet CBT-I offerings that are fully self-contained have proliferated. Examples are Bmedi, Shuti, Sleepio, Sleepful, and Sleepstation. No doubt, the development of CBT-I “apps” was based on the following foundational ideas: (1) CBT-I is a data-driven therapy and thus is easily programmable; (2) data can be captured online and this ensures that the data are prospective and this also allows for automated assessments of adherence; (3) as an online offering, it allows for the implementation of reminders and/or queries and feedback in real time; (4) as an algorithm-based therapy, treatment can be delivered without a therapist and without recourse to state practice regulations; and (5) as an unattended-automated “app”, this form of CBT-I is infinitely scalable. In the final analysis, iCBT-I is a form of self-help but one that substantially differs from written guidelines. In the case of iCBT-I, the core components of treatment are delivered using a wealth of visual aids (video explanations by experts, video patient testimonials, animated white board exemplars, and so on) and therapy implementation requires prospective data collection and algorithm-driven prescriptions.

In what is perhaps the first study of iCBT-I, Ritterband *et al.* assessed an iCBTI intervention in 45 adults who were randomly assigned to an internet condition or wait-list control (WLC)^79^. The treatment condition did significantly better than the comparator. Sleep continuity improved; SL and WASO decreased by 14 minutes (d = 0.63) and 37 minutes (d = 1.15), respectively. TST increased by about 55 minutes (d = 0.72), and SE% increased by 12% (d = 1.1).

Given the above results and the significant positive attributes of the approach, one might wonder “What’s the downside?” iCBT-I offerings may be too automated. That is, they may lack the following: the capacity to screen subjects for the full range of sleep disorders that tend occur comorbidly with insomnia, the ability to conduct differential diagnoses, the capacity to determine when treatment is not indicated, the functionality to refer patients, the flexibility to tailor the prescriptive and educational components of therapy, and the wherewithal to emulate the non-specific factors that fuel the dynamic between patient and therapist. These limitations underscore the need for head-to-head trials between iCBT-I and in-person CBT-I. To the best of our knowledge, only one such study exists: Taylor *et al.* (2017)^80^.

Taylor *et al.*^80^ found, in an RCT of 100 active-duty U.S. Army personnel, that internet and in-person CBT-I performed significantly better than the control condition on prospectively assessed, high-density, self-reported, sleep continuity (sleep diaries). SL decreased by about 10 minutes for the iCBT-I group and by 15 minutes for the in-person CBT-I group (d = −0.4 vs. d = −0.5), number of nighttime awakenings decreased by about 0.8 for both groups (d = −0.5 and d = −0.4), WASO decreased by about 12 minutes for the iCBT-I group and by about 25 minutes for the in-person CBT-I group (d = −0.3 and d = −0.7), and TST increased by about 12 minutes for the iCBT-I group and by about 24 minutes for the in-person CBT-I group (d = 0.17 and d = 0.34). SE% increased by about 7 points for the internet CBT-I group and by about 11% for the in-person CBT-I group (d = 0.5 and d = 0.9). Similar findings were evident for retrospective self-report measures of insomnia severity. The ISI score decreased by about 6 points for the iCBT-I group and by 9 points for the in-person CBT-I group (d = −1.0 vs. d = −1.53). Overall, the effect sizes for in-person therapy were consistently better than internet treatment and the observed magnitudes were similar to those found in civilians. Both studies indicate that iCBTI may be an adequate alternative when in-person or telemedicine is not available. This perspective is supported by several published meta-analyses^81,82^.

***Brief CBT-I (BBT-I).*** One significant development in regard to CBT-I has been the initiative to streamline therapy from its six- to eight-session form to a briefer form entailing two to four sessions^81–83^. This effort has been driven largely by the shortage of CBT-I providers. The idea is that if briefer CBT-I is reasonably efficacious, then it can be adopted by both experienced and perhaps less experienced clinicians so that more patients can be seen by the existing cohort of therapists. In 2006, Germain, Buysse *et al.* introduced the first standardized “short” form of CBT-I, referring to it as brief behavioral treatment for insomnia (BBT-I)^84^. The therapy was proffered not as a replacement for CBT-I per se but as a therapeutic option for when full-scale CBT-I was not available, possible, or indicated on the basis of illness severity, frequency, or chronicity. To date, at least six studies have demonstrated the efficacy of BBT-I as compared with control treatments. One of these studies was undertaken in patients with chronic insomnia (insomnia as a primary disorder)^85^, two were conducted in older adults with chronic insomnia^86,87^, one study was conducted in recovering alcoholics^88^, one study was conducted in patients with PTSD^89^, and one study was conducted in patients with refractory insomnia and residual depression^90^. The average pre-post effect size data from these studies were 0.99 for SL, 1.23 for WASO, and 0.39 for TST. Taken together, these studies, in comparison with the meta-analytic data from Smith *et al.*, suggest that briefer versions of CBT-I appear to be “non-inferior” to “full-dose” treatment. Four of the seven studies, it should be noted, also had follow-up data and these results showed that the pre-to-post treatment gains were maintained over time.

Newer work by Pigeon *et al.* (2017)^91^ tested BBT-I in a sample of veterans in a primary care setting, where subjects were randomly assigned to either a sleep hygiene (SH) condition (n = 14) or a BBT-I condition (n = 13). Treatment consisted of two 20- to 40-minute in-person sessions, two 15- to 20-minute phone sessions, and a 3-month follow-up. BBT-I was found to be superior to SH for SL, WASO, and TST. The pre-post effects for BBT-I were as follows: there was a 28-minute decrease in SL (d = −1.28), a 48-minute decrease in WASO (d = −1.0), and a 20-minute decrease in TST (d = −0.2); SE% increased by 11% (d = 0.95); and the ISI score decreased by 8 points pre-to-post (d = −1.5). The author notes that these changes were not found to be statistically significant at the 3-month follow-up (as compared with the SH condition).

One final comment regarding this particular study, although the comment applies more broadly to BBT-I in general: It should not come as a surprise that presenting complaints in regard to initial and middle insomnia may be resolved within four sessions. If the patient is adherent with sleep restriction, treatment responses (reductions in SL and WASO) should occur within the week following the initial prescription (after session 2). This, putatively, occurs owing to the increase in sleep pressure from the reduction in sleep opportunity. Once TST is compressed and SE% is within a normal range (for example, >85% or 90%), “time in bed” (TIB) titration (systematic sleep extension) may begin. That is, sleep opportunity may be expanded by 15 minutes per week provided that good sleep efficiency is maintained. Herein lies the issue. If the average reduction in TIB is 1 hour, then it will take four sessions for TST to return to baseline levels. In the case of BBT-I, only two sessions are available for titration. Thus, the maximum uptick in TIB is 30 minutes (assuming perfect adherence and linear improvements in sleep ability). This being the case, BBT-I will (on average) necessarily result in reduced TST at treatment end (~30 minutes less than baseline). This is precisely the case in the study by Pigeon *et al.*^91^. In many ways, this is a best-case scenario, as sleep restriction often requires more than a one-hour reduction in TIB. Instead of being a limitation of BBT-I, perhaps it is a clue to when BBT-I is, and is not, indicated. If the initial sleep restriction (prescribed TIB) is 30 minutes or less, BBT-I is indicated. If the initial sleep restriction (prescribed TIB) is greater than 30 minutes, CBT-I is indicated. Whether or not such a differential is possible remains to be explored.

More recently, Ellis *et al.* (2015) proposed an even briefer form of BBT-I, a “single-shot” version that consists of one treatment session and a self-help pamphlet^92^. This form of CBT-I, though designed to treat acute insomnia, may also be useful for populations that cannot easily access multi-session treatment (for example, incarcerated persons). Single-shot CBT-I (ssCBT-I [versus a WLC]) was initially tested in 40 individuals with diagnosed acute insomnia. In regard to sleep continuity and ISI scores, significant group differences were not found at 1-week post-treatment but were resolved at 1 month. Pre-post change score effect sizes (at 1 month) for the ssCBT-I group were as follows: SL, d = 0.71; WASO, d = 0.77; TST, d = 0.28; and ISI, d = 0.64. Pre-post change score effect sizes (at 1 month) for the WLC group were as follows: SL, d = −0.14; WASO, d = −0.31; TST, d = 0.14; and ISI, d = −0.74. These preliminary data suggest that ssCBT-I may be an effective treatment for acute insomnia. Since the original study, there have been three additional studies: one in incarcerated persons^93–95^, one in adolescents with diagnosed anxiety and depression^93–95^, and one in groups^93–95^.

### CBT-I (Alternative/Adjuvant Methods)

***Substituting sleep compression for sleep restriction.*** Perhaps one of the first and most innovative adaptations of CBT-I (more specifically, sleep restriction therapy [SRT]) came with the proposal to substitute sleep compression for sleep restriction. Although the alternative therapy was explicitly proffered for patients with sleep continuity disturbance without daytime sequelae (that is, short sleep patients with normal to high sleep opportunity and low sleep need and ability), it has become common (at least in clinical practice) to use this procedure for patients who are resistant to (and/or non-adherent with) sleep restriction. The method is comparable to sleep restriction in that sleep opportunity is matched to sleep ability (that is, step 1 is to match average TIB to average TST). During sleep compression, unlike sleep restriction (where the “reset” is accomplished on night 1), the reset is achieved over a series of weeks. In the original formulation of this treatment, therapy was discontinued when the patient reached the target SE%. There was no need for systematic sleep extension since it was assumed that the patient had achieved a good match between their sleep opportunity, sleep ability, and sleep need. In more recent iterations, as applied to patients with insomnia disorder, sleep extension remains a component of this treatment regimen. Lichstein *et al.* (2001) first demonstrated this method in a sample of 74 older adults (59 years or older)^96^. Subjects were randomly assigned to one of three groups: relaxation (n = 27), sleep compression (n = 24), or placebo desensitization (n = 23). The sleep compression condition fared better than the placebo group. The pre-to-post change in the subjects who received sleep compression was that SL decreased by about 12 minutes (d = −0.48), WASO decreased by about 34 minutes (d = −0.75), SE% increased by about 8 points (d = 0.55), and TST increased by about 47 minutes (d = 0.20). That study provides preliminary evidence that sleep compression is effective for short sleepers who have low levels of daytime impairment. Less clear in the extant literature is whether patients with insomnia disorder fair equally well when randomly assigned to sleep compression as opposed to sleep restriction.

***Augmenting CBT-I with mindfulness training.*** It can be argued that the single most influential change to CBT-I in the last two decades has been the adoption of mindfulness training. Mindfulness was introduced, in the context of insomnia, by Ong *et al.* in 2008^97^. The stated goal for the adjuvant therapy was to better address sleep-related cognitive arousal. The approach substantially differs from traditional cognitive therapy (CT) in that mindfulness is not focused on disputing, derailing, or disengaging worry or intrusive negative thoughts. Instead, mindfulness is focused on the non-judgmental observation of one’s cognitions, and the desired goal is changing one’s relationship to one’s thoughts as opposed to “fighting” with them. In this way, the process encourages more acceptance. Ong *et al.* (2008) conducted a treatment-development study (using a simple pre-post design) to evaluate the effectiveness of mindfulness meditation as an adjuvant to six-session (90–120 minutes per session) in-person group CBT-I (n = 30)^97^. Significant pre-post treatment reductions were found in the study’s primary outcome variable: TWT (SL + WASO + EMA). The corresponding pre-post effect size for this variable was −1.17. Significant reductions were also found in secondary analyses of the single-component measures, including an 18-minute decrease in SL (d = −0.84), a 25-minute decrease in WASO (d = −0.62), a 5-minute decrease in TST (d = −0.11), and a 9-point increase in SE% (d = 1.1). When assessed in terms of the ISI, a 5-point decrease was observed (d = −1.32). A follow-up study showed that these sleep-related benefits were maintained over 12 months post-treatment^98^. The authors noted that subjects who reported higher pre-sleep arousal and sleep effort at the end of treatment experienced worse long-term outcomes.

In sum, it is clear that individual in-person CBT-I is efficacious for both complicated and uncomplicated insomnia. It is also clear that modified forms of CBT-I (modified by delivery format, by duration of treatment, or with augmenting strategies) produce good treatment outcomes. Less clear is whether or not the various forms of CBT-I are comparably efficacious and, if not, when modified CBT-I is indicated. Clearly, much work remains.

## “Now what (what’s next)?”

At least eight BSM key opinion leaders (KOLs) have published precisely on the issue of “What’s next?”^99–107^ That is, what is the future of CBT-I and what remains to be done in regard to clinical research, standards and policy, dissemination and implementation, and clinical practice? The recommendations proffered by each KOL are presented in Table 2. The number of KOLs referenced in the table exceeds eight because one of the publications^101^ summarized multiple presentations on this topic at a BSM consensus conference in 2011. Following this summary, we address the “what’s next” issue by adopting a Q-and-A format. Each question is followed by an answer that includes what is known and some thoughts about what productive next steps might be.

**Table 2.**
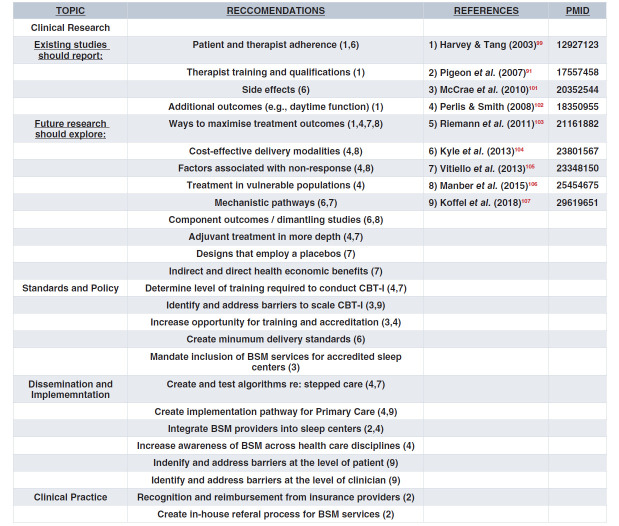
BSM Reccomendations.

The major recommendations, as can be seen in Table 2, pertain to the need for reporting standards for RCTs, investigations into the mechanisms of action related to the various components of CBT-I, studies that seek to improve treatment outcomes, and evaluations regarding how best to disseminate and implement CBT-I, in either primary or tertiary care settings.

### Future directions for clinical research

***Q1: Is there a single component of CBT-I that is essential or that carries the majority of the outcome variance?***

A: Many of the paths forward with regard to “next steps” for CBT-I research have to do with how to better streamline the delivery of this multicomponent therapy. One approach has been to determine which components of therapy are necessary and/or deserve or require more time and which components can be doffed or have less time devoted to them. One method for making such an assessment is via “dismantling studies”. To date, there have been at least two such studies: one by Epstein *et al.* (2012)^108 ^and one by Harvey *et al.* (2014)^109^. Epstein *et al.* found that multicomponent CBT-I may increase remitter rates at treatment end (as compared with single-component SRT and SCT). Harvey *et al.* found that behavior therapy (BT) may lead to faster treatment responses but these gains, in the absence of CT, are not necessarily maintained over time. In contrast, CT led to slower but more sustained treatment gains. As with Epstein *et al.*, Harvey *et al.*^109^ concluded that multicomponent CBT-I is optimal and that both BT and CT contribute uniquely to short- and long-term efficacy. Additional dismantling studies are needed to confirm these first findings.

Another way to streamline therapy is to use a case conceptualization approach. This strategy was developed by Manber and Carney^110^. For example, some cases may involve significant sleep extension (a large mismatch between sleep ability and sleep opportunity) whereas other cases may exhibit more conditioned arousal. The former may benefit more from targeted SRT whereas the latter may require only SCT. Likewise, a case conceptual formulation may highlight other factors such as hyperarousal, sleep-related worry, sleep effort, sleep habits, and medication effects. Conceivably, such a case conceptualization approach may help to isolate the most relevant factors and this, in turn, may allow the clinician to take a more surgical approach to treatment. Although this approach could shorten the length of treatment for patients, it still requires well-trained clinicians who can perform the assessments and provide good case conceptualization.

One final comment: formal risk–benefit analyses should also be conducted in order to assist with the determination of the relative merit of each of the components of CBT-I. For example, although SRT may produce robust clinical gains, it also likely has the worst side-effect profile of the therapies that comprise CBT-I; SRT produces significant acute increases in daytime sleepiness. Thus, it is important for providers to consider, on a case-by-case basis, whether or not SRT can be safely used. Nonetheless, Cheng *et al.* found that SRT has a similar risk profile to CBT-I and, as such, SRT may be a safe alternative^111^.

***Q2: Is patient adherence a make-or-break component and, if so, how do we measure and enhance this?***

A: It is difficult to imagine a scenario where one can expect to recover from chronic illness by doing nothing. Most patients know, or believe, that treatment is required to manage or recover from chronic illness, especially patients who are actively “help seeking”. The real question is a relative one, “Can one do some of the therapy and expect some change?”, especially when (in the short term) treatment adherence requires discomfort and (in the long term) the adoption of behavioral or “lifestyle” changes. Since CBT-I entails both discomfort and behavioral change, it is inevitable that patients wonder “How much of this do I actually have to do to get better?” Given this, one can surmise that some part of the 30% of subjects who are non-responders to CBT-I occurs relative to patients “fractionating doses” (that is, doing some of the prescription some of the time or less rigorous versions of the prescription most of the time). Examples of fractionating doses include, but are not limited to, going to bed at midnight as opposed to a prescribed time of 1 a.m., staying out of bed for 5 minutes as opposed to some meaningful increment of time like 30 minutes, and so on.

To date, only a few studies have empirically assessed non-adherence. Though non-adherence is variably operationalized, the general conclusion is that a large proportion of patients do not closely adhere to SRT and SCT instructions^112–115^. In a 2004 study, using a percentage of subjects compliant with prescribed time to bed (PTTB), researchers found that 51% of subjects during the first four weeks of CBT-I were adherent (no more than 15 minutes earlier to bed than PTTB)^116^. In the worst-case scenario, such data may map directly onto treatment response rates (51% adherence = 51% treatment response). In the best-case scenario, such data suggest that partial adherence can yield good results. Without direct studies on the relationship between adherence and treatment response, one is left to wonder how better-or-full adherence might increase the speed to, or the magnitude of, treatment responses. Clearly, a whole program of research is needed within this arena.

Finally, although there is no consensus on how adherence should be measured, one possible approach, using sleep diaries, is to quantify daily deviations from prescribed TTB and TOB. In this way, one can measure how adherent the individual is to sleep rescheduling by evaluating the mean deviation from the prescriptions per week. This could be done in a continuous or categorical manner. In the case of a continuous measure, the clinician is left to decide “how much is too much” (that is, does a weekly average of 15 or 30 minutes constitute non-adherence?). Whatever threshold is used, weekly sums of the categorical judgments could be used to express the number of days that the patient is adherent^116^. A similar approach could be used to assess the patient’s adherence to SCT. In this case, one might modify how we ask about time spent awake after sleep onset on sleep diaries. (For example, instead of using the generic “WASO”, replace it with WASO-IN and WASO-OUT: time spent awake in the middle of the night in and out of bed.) The deviation between these two measures could also be used to assess adherence continuously or categorically. Adherence may be evaluated not only with sleep diaries but also with wearables, especially those that have event markers.

***Q3: To what extent is CBT-I standardized across RCTs?***

A: The conduct of CBT-I varies greatly from RCT to RCT and likely even more so from practice to practice. To illustrate this point, Table 3a and Table 3b provide the details of CBT-I from four published treatment manuals.

**Table 3a.**
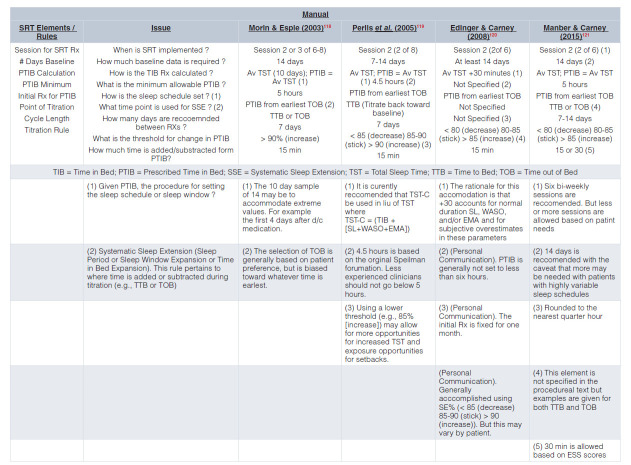
SRT & SCT Rules Sleep Restriction Therapy (SRT) Rules Based on the 1st Four Published Treatment Manuals.

**Table 3b.**
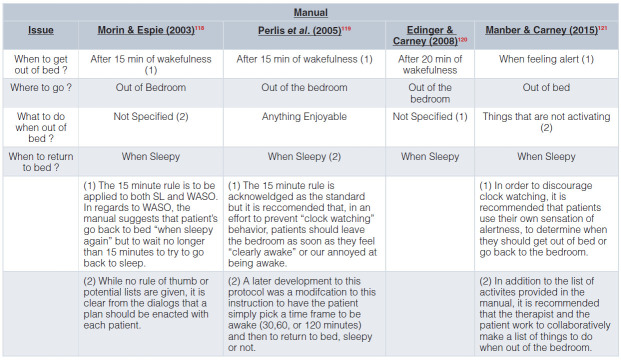
SRT & SCT Rules Stimulus Control Therapy (SCT) Rules Based on the 1st Three Published Treatment Manuals.

In 2015, Kyle *et al.* addressed this issue as it applies to SRT^117^. In this first-of-its-kind systematic review of 88 RCTs, Kyle *et al.* found that of the 85 studies that incorporated SRT, 38% did not report details on any of the five *a priori*–specified parameters of SRT implementation. That is, the studies did not delineate (1) how the minimum TIB recommendation was calculated, (2) the minimum allowable prescription for TIB (for example, 5 hours), (3) the sleep efficiency criteria for titration (for example, >90% add time to TIB), (4) the time unit for titration (for example, ± 15 minutes), or (5) the position of sleep window (for example, chronotype).

Only 7% of studies reported information for all SRT parameters. The most commonly reported single procedural element was the assessment and specification of prescribed TIB (method of assessment and the minimum TIB for sleep restriction)—that is, the specification that sleep diaries were used to calculate average TST and that this value was used to formulate prescribed TIB, generally with a 5-hour minimum. One of the most common adaptations to “Spielman-ian” SRT pertains to prescribed TIB. For example, rather than being set to average TST, TIB is set to average TST + 30 minutes. Given these findings, the investigators conclude that “poor reporting and variability in the application of SRT may hinder progress in relation to evidence synthesis, specification of mechanistic components, and refinement of therapeutic procedures for patient benefit” (p. 83)^117^.

***Q4: Does the issue of optimal dosing for CBT-I need to be revisited?***

A: Many consider this a settled issue, based on one seminal study. In 2007, Edinger *et al.*^122^ evaluated treatment length in patients with “primary” insomnia. The trial was conducted in 86 subjects with sleep maintenance difficulties. Subjects were randomly assigned to one of five conditions (monitor only or active treatment over one, two, four, or eight biweekly sessions). CBT-I included sleep education, SCT, and a modified form of sleep restriction (TIB was restricted to average baseline TST + 30 minutes). Sleep was assessed with prospective sampling of sleep continuity (daily sleep diaries) and actigraphy. In general, the study found no dose-response effects (that is, each stepwise increase in number of sessions did not result in incrementally better outcomes). Instead, one and four sessions appeared to produce the largest effect sizes and to produce the largest percentages of treatment responders. The average pre-to-post effect sizes for one- and four-session CBT-I were as follows: SL, WASO, and TST were 0.5, 1.1, and 0.5 (respectively) for one session and 0.3, 1.3, and 0.3 (respectively) for four sessions. The investigators concluded that four individual biweekly sessions is the optimal treatment for CBT-I. Although that study is the first of its kind, the investigation has several limitations that call into question the conclusion. Of the various concerns that might be raised, none is more important than the fact that *all treatment recommendations were delivered at session 1* and the remaining sessions “consisted of therapist guidance in modifying TIB prescriptions as well as therapist encouragement of treatment adherence” (p. 206)^122^. Since the components of CBT-I are generally delivered over two or three sessions (slowly over time), it can be argued that the question addressed by Edinger *et al.* is more akin to “When CBT-I is delivered in a single session, how helpful are supplementary sessions?” Viewed this way, the question of how many standard sessions are optimal remains to be determined. One other question to ponder in regard to dose is “What should be the goal of CBT-I treatment?” Is the goal just to improve sleep continuity or should there be an additional focus on achieving an optimal amount of TST (presumably a thing that yields as much or more improvement in health outcomes than increasing sleep efficiency). As reviewed above, TST tends to increase following treatment discontinuation (~60% of patients achieve 30 or more minutes of additional TST at 3 to 12 months after six-session CBT-I^21^). When the issue is viewed in this context, perhaps the question is “Can higher doses of CBT-I (additional sessions carried out weekly or spread out over time) speed the latency to this outcome, increase the percentage of patients who achieve this outcome, and/or increase the average gains in TST?”

### Standards and policy issues

***Q5: Is a therapist required for CBT-I? That is, is internet CBT-I good enough?***

A: There can be no question that iCBT-I was developed to reduce wait-list times and to enhance the scalability and reach of in-person CBT-I by providing a more accessible and efficient alternative. As noted above, questions regarding the relative efficacy remain to be exhaustively addressed. Nonetheless, the question posed above remains and has garnered considerably more attention over the last decade given a variety of technological advances (for example, more sophisticated software, better online apps, and the integration of wearable devices). With the proliferation of iCBT-I, it is clear that the algorithms that underlie SRT can be reasonably implemented without a therapist, and alerts and alarms can be used to enhance adherence with scheduled bed and wake times via emails, texts, wearables, and smart speakers. These positive attributes take what is essentially a self-help approach to the next level. At present, there is a wealth of data on the absolute efficacy of the most prominent platforms (for example, SHUTi [Somryst], Sleepio, Sleepful, and Sleepstation). As noted above, these data suggest that iCBT-I is superior to control conditions (for example, wait list, monitor only, or minimal intervention conditions). In contrast, the relative efficacy data (one study at present about iCBT-I vs. in-person, therapist-driven CBT-I) suggest that the effect sizes for in-person therapy are consistently better than those of internet treatment^79^. Although that is only one study, it follows that in-person treatment has a number of advantages, including non-specific dyad/relationship factors and the leveraging of the therapeutic alliance to obtain maximal adherence. At the end of the day, more relative efficacy studies—studies that also vary in therapist expertise—are needed. To date, most RCTs have not been conducted with expert or professional therapists. It is possible, if not likely, that more experienced therapists (1) know how and when to tailor therapy and when not to and (2) are better able to manage patient resistances and to garner adherence. Nonetheless, this remains an empirical question. In the absence of data on this topic, one interpretation of the “as good or better outcomes” with clinical case series data is that when therapists are more experienced, this matters more than the degree to which patients are more complex (that is, have significant comorbidity).

***Q6: Are there practices or procedures that can and should be added to CBT-I overall or for specific clinical populations?***

A: In recent years, it has been suggested that CBT-I be altered to meet the needs of more complex patient groups (that is, patients with illnesses or disabilities that may prevent them from engaging in standard CBT-I). One obvious example is, in individuals unable to complete self-report instruments, could CBT-I be conducted using actigraphs, as opposed to sleep diaries? Another example is the use of sleep compression with bipolar disorder patients to avoid the risk of sleep deprivation–induced mania or counter control for patients with physical limitations that would make full SCT difficult. Yet another modification might be the use of “exercise-based activities” to replace therapist explanations (that is, simile- or metaphor-based illustrations/exemplars) or behavioral experiments to augment or validate therapist explanations^95^. In regard to the former, one often-used explanation is to verbally describe sleep restriction in terms of sleep ability where sleep ability is likened to pizza dough and sleep opportunity and sleep timing to the outer edge of a pie plate (that is, the more of the circumference one attempts to cover, the thinner the dough … which [with enough stretching] simply breaks into pieces). Rather than having this described verbally, some clinical populations may benefit more from actually engaging in (or seeing a video of) the task. Although such adaptations are innovative and developed with laudable intentions, no studies to date show that such adaptations produce significantly better results in any subpopulation of patients with insomnia.

***Q7: Should we increase and diversify those who provide CBT-I?***

A: The problem of “too few providers” is not a new one for the BSM field^92,93^. Steps have been taken over the course of the past two decades to increase the size of the BSM workforce and the availability of CBT-I^100,107^. Initial efforts focused on (1) the creation of a certification process for clinically licensed clinicians with a PhD, PsyD, or MD (initially via the American Board of Sleep Medicine and later via the Board of Behavioral Sleep Medicine); (2) the development and provision of continuing education (CE) and continuing medical education (CME) CBT-I training; (3) the establishment of a society dedicated to CBT-I and BSM; and (4) the publication of professional articles regarding the need to make (and logistics of making) CBT-I universally available^100,102^. Two of the above efforts (the establishment of a BSM society and CE/CME training) had, as a keystone issue, extending training and certification to master-level clinicians (nurse practitioners, physician assistants, social workers, occupation therapists, and so on). Although these efforts have been successful, the provider shortage has persisted. Part of the problem has to do with reimbursement for CBT-I (that is, reimbursement for the intervention in its evidence-based form, by whom, in what venue, and so on). Some argue that, once this issue is resolved, it will serve as the impetus for clinicians from diverse fields to seek out training and certification. Although universal reimbursement may serve to increase the availability of CBT-I, a dedicated lobbying effort will be needed to secure this and much planning will be required to ensure that the resources required to meet the new demand are available (that is, that adequate training, test prep, credential review, and exam administration facilities are available).

One additional cautionary note: embracing a diversity of disciplines will come with a multitude of logistical problems that need to be anticipated and resolved *a priori*^100,105^. For example, training clinicians with different backgrounds will require either courses that are very comprehensive or courses that are delivered by discipline. Both prospects are somewhat daunting and the effort to secure CE/CME accreditation for multiple professions may be even more daunting. Nonetheless, good models exist. Several organizations—including the University of Rochester/Pennsylvania (Perlis, 2005), the DOD (Brim, 2009), the Veterans Administration (Manber, 2010), Ryerson University (Carney, 2015), Oxford University (Espie, 2016), and the University of Arizona (Taylor, 2020)—have successfully developed and deployed multidisciplinary, non-sleep specialist training. However, it should be noted that no amount of CE/CME training can create a supply of clinicians who can meet the demand. Perhaps the demand will be met when training in sleep medicine and BSM becomes a staple of all medical and graduate school curricula. For example, the medical school and graduate school curricula should include at least two core lectures: (1) a lecture on the health consequences of sleep disorders and how medical disease and sleep disorders are often reciprocally related and (2) one lecture on the assessment and treatment of sleep disorders. These could be made available by the American Academy of Sleep Medicine or the Society of Behavioral Sleep Medicine as videos or live-streamed lectures. Until such a time comes to pass, the use of online screeners, apps, and BBT-I can and must serve as the base of a stepped care pyramid, freeing credentialed clinicians to serve those who present with more complexity or greater illness severity or have failed on lower rungs of the pyramid.

***Q8: Should CBT-I be prescribed and, if so, by whom?***

A: Although such a question may strike many of us as strange (referral is one thing but prescription is another), the issue has been raised in the context of iCBT-I. Given the recent U.S. Food and Drug Administration approval of “Somryst” (a version of SHUTi), it follows that those with prescriptive authority (for example, MDs, NPs, and DOs) will soon be, if they are not already, in the position of prescribing this internet digital therapeutic. To many, this is a concerning development, especially as a professional practice issue. Leaving aside guild considerations, this development can be viewed as a positive thing provided that (1) a decision algorithm (one that is subject to modification over time) is in place regarding who is appropriate for iCBT-I and (2) an adequate monitoring system is in place to know when treatment is not effective and referral to specialty care is required.

### Dissemination and implementation issues

***Q9: Should some form of CBT-I/BSM services be offered in primary care?***

A: The prevalence of insomnia in primary care is high; up to 50% of primary care patients report at least occasional insomnia, and 19% report chronic insomnia^123–126^. According to one epidemiologic study^127^, of the patients who consulted a medical professional about insomnia, 82.7% consulted their general practitioner. These data suggest that there is certainly an opportunity and a need to provide first-line interventions in primary care. Given the need and opportunity, the next questions are (1) is insomnia currently being treated in primary care (and, if so, how), (2) is it feasible to offer standard CBT-I in primary care, and (3) what patients should receive treatment and who should provide care?

In regard to the first question, a qualitative study on the management of insomnia in primary care was conducted by Davy *et al.* (2015)^128^. The study included interviews of both practitioners and patients. It was reported that the focus of treatment was primarily on comorbidities and not on insomnia. For the patients who received targeted treatment, it typically involved some combination of SH “tips” and the prescription of hypnotics^128^. Both practitioners and patients expressed the need for more treatment options and training on insomnia. In another study by Vidal-Thomas *et al.* (2017), 138 primary care nurses were surveyed, and only 11% of nurses were found to have received any training in sleep interventions during the previous 5 years {Vidal-Thomas, 2017 #216}. Taken together, these studies suggest that insomnia is not well managed in primary care (that is, the modal treatment options are not first-line therapies^16^), and when it is, many of the practitioners do not have adequate knowledge or training to offer CBT-I as an option.

In regard to the second and third questions, quantitative research has been conducted with abbreviated versions of CBT-I (four to six sessions) as provided in primary care venues, where most of the interventions were in a group format^129,130^. The results from these studies suggest that abbreviated CBT-I can be effective when used to treat insomnia symptoms in primary care. For example, in a series of studies, Espie *et al.* (2001, 2007, 2013)^131–133^ showed that 6-week, nurse-administered, group CBT-I produced significant improvements at post-treatment. The most recent study showed significant increases in SL (d = −0.15) and WASO (d = −0.31) and a significant decrease in ISI scores (d = 0.95). Similar studies have shown that these effects can be sustained for 3 to 12 months after treatment^134–136^. The observed effects, however, are modest in comparison with meta-analytic norms for standard, full-length CBT-I. This suggests that more work is needed to determine how CBT-I can best be implemented in the context of primary care, not only to optimize outcomes but also to ensure that those who require more extensive evaluations and treatment are either triaged early on, or are referred on, as needed. One approach to triage could be based on initial insomnia severity, prior use of hypnotics, the co-incidence of short sleep, and a measure of the treatment intensity required for therapy^137–140^. In regard to the last of these, if the mismatch between sleep opportunity and sleep ability is greater than “X” (for example, 45 minutes), such cases might be better managed by clinicians with an established expertise in CBT-I (master-level clinicians such as credentialed and experienced therapists with a track record of supervisory, educational, or research experience).

***Q10: Should CBT-I / BSM services be offered in tertiary care?***

A: Although sleep disorder centers seem an ideal venue for the delivery of CBT-I and BSM services, it appears that only a minority of centers have “in house” experts. While implementation of such services can be daunting, in the absence of an accreditation mandate, this situation is unlikely to be resolved in the near future^102^. Apart from sleep centers, other tertiary care venues, including psychiatric clinics, cancer centers, pain management services, and gerontology practices, are natural homes for CBT-I and BSM services. Not only do these venues have disproportionate rates of insomnia in their clinical populations, but such practices often are accustomed to making available health psychology and behavioral medicine interventions. In the future, systematic attempts should be made (at both the individual and professional levels) to explore matching tertiary care centers to available BSM/CBT-I specialists, which could be an initiative taken up by the Society of Behavioral Sleep Medicine.

### Clinical practice issues

***Q11: What is the role of technical language in clinical practice?***

Put differently, “Is it helpful to use specific technical terms when conducting CBT-I or do use of these terms lead to some form of inoculation (that is, familiarity with the names of the various therapies makes them seem passé)?” Furthermore, it has been suggested that the technical terms for the component therapies of CBT-I do not properly convey to the patient the essence of what the technique actually does, which sometimes can be anxiety-provoking. Is it possible that adoption of different terminology positions the patient to be more receptive and therefore more likely to be adherent? Toward this end, sleep restriction might be better referred to as TIB restriction, sleep efficiency training, or sleep rescheduling; SCT might be better referred to as reassociation training or reconditioning therapy; and SH might be better referred to as a review of unhelpful sleep behaviors, cataloguing behaviors that help and hurt sleep, or sleep–wake optimization. To the best of our knowledge, no empirical work has been conducted on this issue. In the absence of empirical work, clinicians are encouraged to consider whether adoption of this recommendation might be useful.

***Q12: What is the role of data in clinical practice?***

In most clinical practices, such a question represents whether or not to engage a kind of monitoring that is not part of the therapeutic process (monitoring of treatment outcome or patient satisfaction or both). In the case of CBT-I, regular symptom monitoring is standard. If there are no data (daily assessments of sleep continuity), there’s no CBT-I. That is, without high-density prospective sampling of sleep continuity (use of sleep diaries), the treatment provided is not evidenced-based CBT-I. Perhaps the real question here is “What type of data is required for best practice CBT-I?” This question was addressed as it applies to clinical research at the Pittsburgh Consensus Conference in 2005^141^ and has been repeatedly engaged as both a practice and research issue in the assessment-of-insomnia chapters in *The Principles and Practice of Sleep Medicine*^142^. Although it goes without saying that daily sleep diaries are required (and that there has been a recommendation for a consensus version of how this should be standardized^143^), most would agree that best practice CBT-I also requires the weekly monitoring of *sleepiness* and *depression symptoms*. To this, many add the Dysfunctional Beliefs Associated with Sleep Questionnaire (DBAS) as part of their intake evaluation and the ISI as a way to measure pre-post change. Going forward, one might ask “Are there other assessments that could and should be undertaken?” Broadly speaking, what may be next is the regular monitoring of daytime function, both globally and specifically. Global assessments may be accomplished with instruments like the Functional Outcomes of Sleep Questionnaire (FOSQ-10)^144^. Specific assessments may be related to the assessment of comorbid illness. For example, if the patient presents with both insomnia and chronic pain, perhaps it is incumbent on us to gather at least pre-post measures of pain. Although one cannot measure all of the comorbidities that occur with insomnia, it may be productive to measure the ones that are thought to be functionally related to sleep and that are of relevance to the patient. Beyond this, data acquisition may be useful, and someday required, for routine program evaluation for the purposes of securing third-party reimbursement. If and when such evaluation becomes standard, CBT-I clinicians will be well positioned to gather such data as it is (and has always been) part of the standard of practice. Perhaps this will be even easier as internet-based tools and smart phone apps (screeners, diaries, and so on) replace paper-and-pencil assessments in patient care. A number of data capture packages have replaced paper-and-pencil assessment, including Sleep Coach, Circady-Pro, Consensus Sleep Diary, and Hypknowledge. The hope is that these, and other data capture packages, fully replace paper-and-pencil methods. Finally, it may benefit CBT-I practice to obtain measures of patient/consumer satisfaction.

***Q13: Should actigraphy, wearables, or nearables be a standard part of assessment for CBT-I?***

A: In a word, “No”. There is no evidence that CBT-I can be successfully conducted with this form of sleep continuity data. More than likely, any effort to replace self-report measures with wearables will require research to determine how concordant such data are with day-to-day patient perception of illness severity. As such, it would be helpful if there were trials that compared device with sleep diary data. If the data are substantially discordant, the initial TIB prescription or titration recommendations (or both) will likely make little sense to the patient and it is likely they’ll be less adherent with devices, as opposed to diary-driven therapy. In the case of treatment outcome, what good is it if a patient exhibits improvement based on devices and does not perceive such improvement (as assessed with self-report measures)? Ultimately, the conduct of CBT-I must change the experience of insomnia or how one tolerates sleep continuity disturbance (or both). For an additional discussion about CBT-I data, see Q6. For a more elaborate discussion regarding the role of the objective measures of insomnia and their utility for assessment and treatment, see Chapter 208 in the 7th edition (2022) of the *Principles and Practice of Sleep Medicine*^145^; for more information on actigraphy, wearables, and “nearables”, online lectures are available via the University of Arizona’s Behavioral Sleep Medicine Lecture Series^146,147^. Nonetheless, devices may serve as useful adjunctive measures. Device data can serve as a check on patient adherence (that is, to confirm whether patients are adhering to the prescribed sleep schedule or whether patients are practicing SCT or both). Devices can also be helpful in cases where (1) there is a suspicion of sleep/wake irregularities that might be related to circadian rhythm disturbances (for example, irregular or free running patterns), (2) the detection/confirmation of paradoxical insomnia is required, or (3) self-report data cannot be acquired (for example, in children, in individuals with severe cognitive impairments, or in patients with dementia). Finally, “wearables and nearables” may be useful for enhancing compliance and rapport: compliance because of the “nanny cam effect” (patients report more honestly because they feel surveilled) and rapport because of a halo effect (when the therapist effectively integrates the use of the device/data into their treatment, it tends to enhance patient confidence that “they are in the right place with the right person”).

***Q14: Is combined therapy for insomnia ever indicated (CBT-I plus meds)?***

A: A variety of RCTs have evaluated how treatment outcomes vary when sedatives (for example, temazepam or zolpidem) are prescribed in conjunction with CBT-I^22–24,148^. Combined treatment and monotherapy with CBT-I have been found to produce equivalent gains in the short term (during acute treatment)—that is, similar gains in regard to sleep initiation and maintenance (diary measures of SL, WASO, and TST), similar gains on multifactorial measures of insomnia severity (ISI), and similar percentages in regard to the subjects who achieve treatment responses. In contrast, there is some evidence that the long-term durability of CBT-I may be lessened in patients co-treated with hypnotics (e.g., Zolpidem) as they tend not to exhibit the increases in TST following treatment discontinuation^149^. Given this profile, one might wonder under what circumstances combined treatment might ever be indicated. The possibilities include (1) when time to a treatment response needs to be accelerated^24^ and (2) when the short-term iatrogenic effects of CBT-I need to be blocked or attenuated. Some have argued that one or both of these considerations can be better managed with the short-term use of stimulants (for example, modafinil once a day before noon or twice a day along with CBT-I: first four sessions of SRT). Although some data suggest that this strategy blocks iatrogenic sleepiness^116^, no evidence has shown that co-treatment with stimulants speeds the latency to treatment response or maintains the long-term benefits of monotherapy with CBT-I. Clinically, both strategies seem to be reasonable alternative approaches for the management of insomnia, when indicated. Though not precisely within the scope of this section, the medical treatment of insomnia (alone or in combination with CBT-I) carries with it concerns about side effects (for example, residual sedation and the risk for psychological or physiological dependence). One advantage of combined therapy is that these potential adverse outcomes are minimized given the intentionally shorter treatment regimens (for example, co-treatment with sedatives or stimulants for 4 to 8 weeks).

***Q15: Is a stepped care approach feasible and what “steps” are necessary to make it so?***

A: Given the “mismatch” between the high demand for insomnia treatment and low supply of BSM providers^105,106,150^, some have suggested that the problem can be resolved (or partially addressed) by adopting a “stepped care” approach. In general, a stepped care approach includes multiple alternatives for treatment that begin with the most cost-, time-, and resource-efficient option of the evidenced-based options, where follow-up steps are clearly delineated. The decision regarding what rung of stepped care to begin with should also be informed by the patient’s preference and clinical profile (that is, illness severity, amount of comorbidity, and degree to which the patient requires guidance and support). Care is then incrementally increased on the basis of the level of response to treatment at each level and assessed needs regarding what further work is required. Another way to implement stepped care (and thereby proffer low-dose treatment) is to attempt monotherapy with SRT or SCT or even SH when appropriate. It seems to us that stepped care can be successful only with a component that is dedicated to surveillance. That is, stepped care requires that some clinician be responsible for the initial assessment, the monitoring of outcome, and the referral of the patient from one level to the next.

Espie (2009)^150^, for example, proposed a stepped care approach to insomnia care that begins with self-administered CBT-I (via book or online). Depending on how patients fare, the patients are then referred to group CBT-I delivered by a trained practitioner (nurse, social worker, or other health professional), individual or group CBT-I delivered by a psychology graduate student, individual CBT-I delivered by a clinical psychologist, and then finally, for the most complex or severe cases, individual CBT-I delivered by a BSM specialist. This is just one example of a stepped care model. Although this approach focuses on who is providing the treatment, other “steps” in the model could also focus on how long (dose/number of sessions) and where CBT-I is delivered. Although the Espie approach is an eminently reasonable one, effective implementation would likely work best given the following: (1) adequate assessment and referral at step 1, (2) the monitoring of cases for treatment response and non-response, (3) the availability (within a system) of all the recommended steps, and (4) a plan for managing inoculation effects when multiple steps are required. Additionally, care should be taken so that the patient clearly understands (from the outset) that non-response at any step does not signal complete failure but rather an opportunity for further assessment and more refined treatment.

***Q16: How can we make CBT-I services reimbursable?***

A: The economic burden of insomnia includes both direct costs (for example, medical appointments, consultations, and products) and indirect costs (absenteeism at work and reduced productivity). Daley *et al.* (2009)^151^ estimated the cost of insomnia in Quebec to be about $6.6 billion (Can), and the largest proportion of expenses were related to indirect costs (for example, work absences and decreased productivity). Many believe that insomnia treatment would greatly curtail such costs, but at present only a few studies demonstrate that this is the case^152–155^. Assuming that treatment does result in substantial health-care cost savings, the widespread implementation of insomnia treatment remains beyond reach. This is true for two reasons: (1) there is a paucity of CBT-I providers, which has led to a supply/demand problem, and (2) CBT-I services are not universally reimbursable. Currently, the reimbursement issue is a battle being waged with third-party payers (that is, insurance companies, by city, by state). So long as this scenario persists, the least that can be done is to share information. For example, who are the third-party payer contacts (by company, by city, by state)? When applications are successful, what information was provided? Perhaps such documents could be warehoused by our professional societies if not developed by them. Beyond this, a more strategic approach could and should be adopted, one where individuals, organizations, or societal agencies take up the mandate and lobby for changes to the current reimbursement structure. Although such efforts may require professional advocates, it may also be productive to seek out and enleague high-profile spokespeople who have an expressed interest in sleep health.

***Q17: To what extent is clinical practice “evidence-based practice”?***

A: If there is as much variance in RCTs as there is (for example, as summarized by Simon *et al.*^117^), one can well imagine that the variability in clinical practice is substantially higher. Our sense is that CBT-I is very robust and that the coverage of the basics will yield positive outcomes. Nonetheless, in any given practice, how have the nuances of treatment delivery by individual or application (or both) varied from what is evidenced-based? Are the changes for the better or worse? Because CBT-I is data-driven, every practice/practitioner is in a position to ask and answer such questions and should. Although some day such program evaluation may be required for reimbursement, for the near future such reviews will help each clinician identify their strengths and weaknesses. Perhaps such reviews could be undertaken in the context of CE and be conducted as a professional consultation such that the data reviews serve to support accreditation.

***Q18: What should be done when CBT-I does not work (for the 30% who are treatment non responders?***

A: The first issue here is what constitutes treatment non-response. The second issue is what to do about it. Typically, treatment response is defined as one or more of the following: (1) 50% improvement on any given metric, (2) ISI score changes of more than 8 points, (3) changes in full ISI scores from clinical to non-clinical ranges, and (4) sleep efficiencies of more than 85%; and so on^156,157^. Ultimately (and perhaps most relevant for clinical practice), treatment response is when the patient says they are better and they are satisfied with the treatment results. If non-response refers to individuals who have had a therapeutic response but not at (or above) desired thresholds, this raises the question (assuming good compliance), “What adjuvant treatment might be required to enhance the response?” Depending on further assessment, such adjuvants might include phototherapy, additional cognitive work, mindfulness, and acceptance-based therapies. If, however, non-response refers to individuals who have not had a treatment response (no change or only minimal change), then many would agree that non-response represents a part of the therapeutic process (that is, “treatment is assessment”); that is, treatment non-response should cause one to re-initiate the assessment process. To our way of thinking, the first step is to evaluate whether the patient has been adherent with the therapy prescriptions and whether the dose of CBT-I (particularly in regard to SRT) was high enough. Beyond these things, it may be that predisposing or precipitating factors remain in play and require direct therapeutic interventions. More research is needed regarding systematic and evidence-based approaches to the management of treatment non-response.

## Concluding remarks

Much remains to be systematically studied and much remains to be elucidated. For example, little is known about how treatment response varies by age, sex, race, and comorbid illness and how such variability might be addressed by the modification of standard CBT-I. This, however, does not detract from the fact that “off the shelf” in-person CBT-I is long past ready to be “scaled up”. The challenges at present are how to increase patient and clinician awareness about CBT-I, how to make CBT-I readily available, and how to make CBT-I universally reimbursable. Put differently, the challenge is how to make CBT-I half as available as pharmacologic treatment. Ultimately, this issue may need to be framed in economic terms: “Should change be driven by addressing the supply or the demand side of the equation?” The most common perspective is that the supply side must be addressed first. That is, it would be virtually unconscionable to escalate the demand for CBT-I knowing that the need cannot be met. The counterargument is that without high demand (and the socioeconomic pressures that come with this), there is not enough of an impetus for the changes required to allow for scaling. Perhaps the answer is that both sides of the equation need to be addressed concurrently. Daunting as this may be, it is our hope that the issues and ideas raised in this article will allow each of us to identify a piece of the mission to make one’s own. Furthermore, it is our hope that our professional societies will embrace and guide individual efforts so that all patients who want CBT-I can receive evidence-based CBT-I.
